# Bta-miR-223 Targeting CBLB Contributes to Resistance to *Staphylococcus aureus* Mastitis Through the PI3K/AKT/NF-κB Pathway

**DOI:** 10.3389/fvets.2020.00529

**Published:** 2020-08-21

**Authors:** Shuo Han, Xinli Li, Juan Liu, Ziwen Zou, Lin Luo, Rui Wu, Zhihui Zhao, Changyuan Wang, Binglei Shen

**Affiliations:** ^1^College of Animal Science and Veterinary Medicine, Heilongjiang Bayi Agricultural University, Daqing, China; ^2^Heilongjiang Provincial Key Laboratory of Prevention and Control of Bovine Diseases, Heilongjiang Bayi Agricultural University, Daqing, China; ^3^Agricultural College, Guangdong Ocean University, Zhanjiang, China; ^4^Heilongjiang Provincial Key Laboratory of Efficient Utilization of Feed Resources and Nutrition Manipulation in Cold Region, Heilongjiang Bayi Agricultural University, Daqing, China

**Keywords:** *Staphylococcus aureus*, bovine mastitis, bta-miR-223, CBLB, resistance regulation mechanism

## Abstract

Bovine mastitis is an inflammatory condition of the mammary gland often caused by (*Staphylococcus aureus*) *S. aureus* infection. The aim of this study was to identify mastitis-related miRNAs and their downstream target genes, and therefore elucidate the regulatory mechanisms involved in disease progression and resistance. Three healthy and three mastitic cows were identified on the basis of the somatic cell count and bacterial culture of their milk, and the histological examination of udder tissues. High-throughput RNA sequencing and bioinformatic analyses revealed that 48 differentially expressed miRNAs (DEMs) in the mastitic udder tissues relative to the healthy tissues. Among 48 DEMs, the expression level of bta-miR-223 was the most up-regulated. Overexpression of the bta-miR-223 in Mac-T cells mitigated the inflammatory pathways induced by *S. aureus*-derived lipoteichoic acid (LTA). The Cbl proto-oncogene B (CBLB) was identified as the target gene of bta-miR-223, and the direct binding of the miRNA to the CBLB promoter was confirmed by dual luciferase reporter assay using wild-type and mutant 3'-UTR constructs. Furthermore, overexpression of *CBLB* in the LTA-stimulated Mac-T cells significantly upregulated PI3K, AKT, and phosphorylated NF-κB p65, whereas *CBLB* knockdown had the opposite effect. Consistent with the *in vitro* findings, the mammary glands of mice infected with 10^8^CFU/100 μL *S. aureus* showed high levels of CBLB, PI3K, AKT, and p-NF-κB p65 48 h after infection. Taken together, bta-miR-223 is a predominant miRNA involved in mastitis, and bta-miR-223 likely mitigates the inflammatory progression by targeting CBLB and inhibiting the downstream PI3K/AKT/NF-κB pathway.

## Introduction

Bovine mastitis is an inflammatory condition of the udders that is caused due to bacterial infection or physical injury (1). It reduces the quality of milk and requires a prolonged treatment regimen, resulting in significant economic losses for the farmers (2, 3).

*S. aureus* is the major causative agent of mastitis, and compared to other bacterial infections, that of *S. aureus* is characterized by immunosuppression, chronic symptoms, and a long incubation period (4). MicroRNAs regulate mammalian innate and adaptive immune responses at the post-transcriptional level by binding to target mRNAs (5).

Bovine mastitis-related miRNAs have been isolated from the blood (6), exosomes (7), mammary epithelial cells (8), and mammary gland tissues (9) of infected animals. Fang et al. established a model of bacterial mastitis in Chinese Holstein cows by injecting *S. aureus* directly into the mammary glands, and identified 77 differentially expressed miRNAs in the inflamed vs. healthy mammary tissues (10). Likewise, Le et al. identified 11 up-regulated and three down-regulated known miRNAs in the milk exosomes of mastitic cows compared to healthy controls (11). Ju et al. also analyzed the miRNA expression profiles of healthy vs. *S. aureus*-infected mammary glands, and detected 277 known and 49 novel miRNAs (12). Luoreng et al. sequenced the mammary gland tissues of cows infected with *E. coli* or *S. aureus* and identified 1,838 miRNAs, including 580 known and 1,258 novel miRNAs (13). MiR-223 plays a key role in the host defense against *S. aureus* infection by inhibiting *CXCL14* and *KIT* (10), and also regulates the proliferation and invasion of human breast cancer cells by targeting *Caprin-1* (14). In addition, miR-223 sensitizes triple negative breast cancer stem cells to *TRAIL*-induced apoptosis by targeting *HAX-1* (15), and inhibits breast cancer progression by targeting *STIM1* (16) and *EGF* (17) pathways, as well as the proliferation of endometrial cancer cells by targeting *IGF-1R* (18).

The aim of our study was to screen for the differentially expressed miRNAs (DEMs) between healthy and mastitic mammary tissues in order to identify the miRNAs associated with the resistance mechanisms against *S. aureus*-induced mastitis, and elucidate the underlying mechanisms.

## Materials and Methods

### Histopathological Examination of Mammary Tissues

Eight Chinese Holstein cows intended for slaughter were provided by the Heilongjiang Yuda Animal Husbandry, China. Fifty-milliliters milk samples were collected from the four milking areas of each cow into sterile centrifuge tubes, and the animals were then euthanized. The udders were disinfected thrice with 75% alcohol, and the skin was peeled using pre-cooled sterile surgical scissors. Pre-cooled sterile scalpels were then used to cut 40 pieces of mammary tissues measuring 0.3 × 0.3 × 0.5 cm. After rinsing thrice with PBS, 10 tissue pieces per animal were immersed in 4% paraformaldehyde, and the remaining pieces were snap frozen in liquid nitrogen and stored at −80°C. The fixed tissues were dehydrated, clarified, embedded in paraffin, and stained with hematoxylin and eosin as per standard protocols. The stained mammary gland sections were then observed for the morphological changes associated with mastitis.

### Bacterial Identification in Milk Samples

The somatic cell counts (SCC) of the 32 milk samples were determined using a cell counter (Thermo Fisher Scientific, USA). Briefly, SCC of 100,000/mL indicated healthy udder, 200,000–500,000/mL indicated subclinical mastitis, and >1,000,000/mL indicated clinical mastitis (19, 20). The milk samples were then inoculated into LB broth and cultured overnight at 37°C. Ten microliters of each suspension was plated on LB agar and cultured at 37°C for 24 h. The individual colonies were picked and subjected to Gram staining to distinguish between gram positive/negative cocci or bacilli. The colonies initially identified as cocci were further sub-cultured into LB broth and blood agar, and finally differentiated as streptococci or *S. aureus* on the basis of their colony morphology, and the catalase and coagulase tests. The colonies identified as *S. aureus* were inoculated into LB broth and incubated overnight at 37°C.

### miRNA Sequencing and Bioinformatic Analyses

Total RNA was extracted from 50 mg frozen udder tissue per animal using the 5 min rapid RNA extraction kit (HiGene, China) as per the manufacturer's instructions. The miRNA libraries were constructed using the small RNA sample premix kit (Illumina, USA), and sequenced using HiSeq2500. The miRNA profiles of three healthy cows (accession numbers are SRR8185411, SRR8185412, and SRR8185413) and three mastitic cows (accession numbers are SRR8185414, SRR8185415, and SRR8185416) were deposited in the SRA database of NCBI. The known and novel miRNAs were identified using the Bowtie (http://bowtie-bio.sourceforge.net/index.shtml) and miRDeep2 (https://www.osc.edu/book/export/html/4389) programs. EBseq (21) was then used to screen for the DEMs using corrected *p*-value (Benjamini-Hochberg method) < 0.05, false discovery rate (FDR) ≤ 0.05 and | log_2_^(FC)^ | ≥ 1 as the screening criteria. The target genes of the DEMs were predicted using TargetScan (http://www.targetscan.org/vert_72) and miRWalk (http://mirwalk.umm.uni-heidelberg.de/), and functionally annotated to GO terms and KEGG pathways using DAVID (https://david.ncifcrf.gov/).

### Establishment of *in vitro* Mastitis Model and Transfection

Mac-T cells (Laboratory of Animal Stress Regulation Mechanism of Heilongjiang Bayi Agricultural University) were stimulated with 1, 10, 20, and 40 μg LTA for 3, 6, 12, and 24 h to induce inflammation. The bta-miR-223 mimic (5′-UGUCAGUUUGUCAAAUACCCCA-3′), mimic negative control (NC; 5′-CAGUACUUUUGUGUAGUACAA-3′), inhibitor (5′-UGGGGUAUUUGACAAACUGACA-3′), and inhibitor NC (5′-CAGUACUUUUGUGUAGUACAA-3′) sequences were purchased from Shanghai Sangon Biotech, China. The cells were transfected with 30 pmol/ml of the above oligonucleotides using RFect transfection reagent (Changzhou Biogenerating Biotechnologies, China), and the fluorescence intensity was checked at 0, 12, 24, 36, and 48 h post-transfection. In addition, the Mac-T cells were also transfected with The 4 μg/mL *CBLB* overexpression and interference vectors (Hedgehogbio Biotechnology, China) using Lipofectamine 3000 (Thermo Fisher Scientific, USA), and the fluorescence intensity was checked after 0, 24, 48, and 72 h of transfection. The expression/silencing efficiency was assessed by RT-PCR and Western blotting.

### Establishment of *S. aureus*-Induced Mastitis Model in Mice

After 1-week of adaptive feeding, 108 8-weeks-old BALB/c mice (81 females and 27 males; Changsheng Biotechnology, China) were set up for mating with three females and one male per cage. The pregnant mice were divided into the PBS, and the 6, 12, 24, and 48 h *S. aureus*-infected groups (*n* = 6 each). The mice were separated from their pups 7 days postpartum and weaned for 6 h. The standard *S. aureus* CMCC(B)26003 strain (Biobw Biotechnology, China) suspension was prepared by inoculating 5 μL of the glycerol stock in 200 ml LB broth and culturing overnight at 37°C till the bacterial load was 10^9^CFU/mL. After anesthetizing the mice with ether, the fourth pair of nipples were disinfected with 75% alcohol and gently lifted with sterilized surgical forceps. Around 100 μL *S. aureus* suspension or PBS was slowly injected into the mammary gland along the milk duct. The mice were put back into the cages and euthanized after 6, 12, 24, and 48 h. The tissue under the fourth nipple pair was dissected, and mammary glands measuring ~0.2 × 0.2 × 0.2 cm were fixed and embedded in paraffin for routine histological staining. In addition, 20 mg tissue per mouse was snap frozen in liquid nitrogen for RNA and protein extraction.

### RT-PCR

The miRNAs were reverse transcribed using a first-strand cDNA synthesis (stem-loop method) kit (Sangon, China) and specific primers (Table S1). RT-qPCR was performed using TB green® premix Ex Taq™ (TAKARA, Japan) and the primer sequences are listed in Table S2. The primer sequences specific for bovine *TNFA, IL1B*, and *IL6*, bovine *CBLB*, sp1 transcription factor (*SP1*), conserved helix-loop-helix ubiquitous kinase (*CHUK)* and interleukin 6 signal transducer (*IL6ST)*, and murine *TNFA, IL6*, and *IL1B* are listed in Tables S3–S5, respectively.

### Western Blotting

Total protein was extracted from the suitably treated cells and murine tissues using the RIPA lysis buffer. Western blotting was performed as per standard protocols using antibodies against CBLB, PI3K, AKT, NF-κB, and p-NF-κB p65 (Proteintech, USA).

### Dual Luciferase Assay

HEK-293T cells (Procell Life Technology, China) were co-transfected with 4 μg/ml wild-type or mutant *CBLB*-3'-UTR along with bta-miR-223 mimic or mimic NC using Roche X-tremeGENE HP DNA transfection reagent (Promega, USA). The dual-glo luciferase assay system kit (Promega, USA) was used to detect the fluorescence activity.

### Statistical Analysis

The relative miRNA and mRNA expression levels were measured using the 2^−ΔΔCt^ method. Image J software was used to analyze the gray value of protein bands. One-way ANOVA analysis was performed using SPSS 19.0, and the data was shown as mean ± standard deviation. *P* < 0.05 was considered statistically significant.

## Results

### Screening and Identification of Healthy and Mastitic Cows

**Figure 1:**
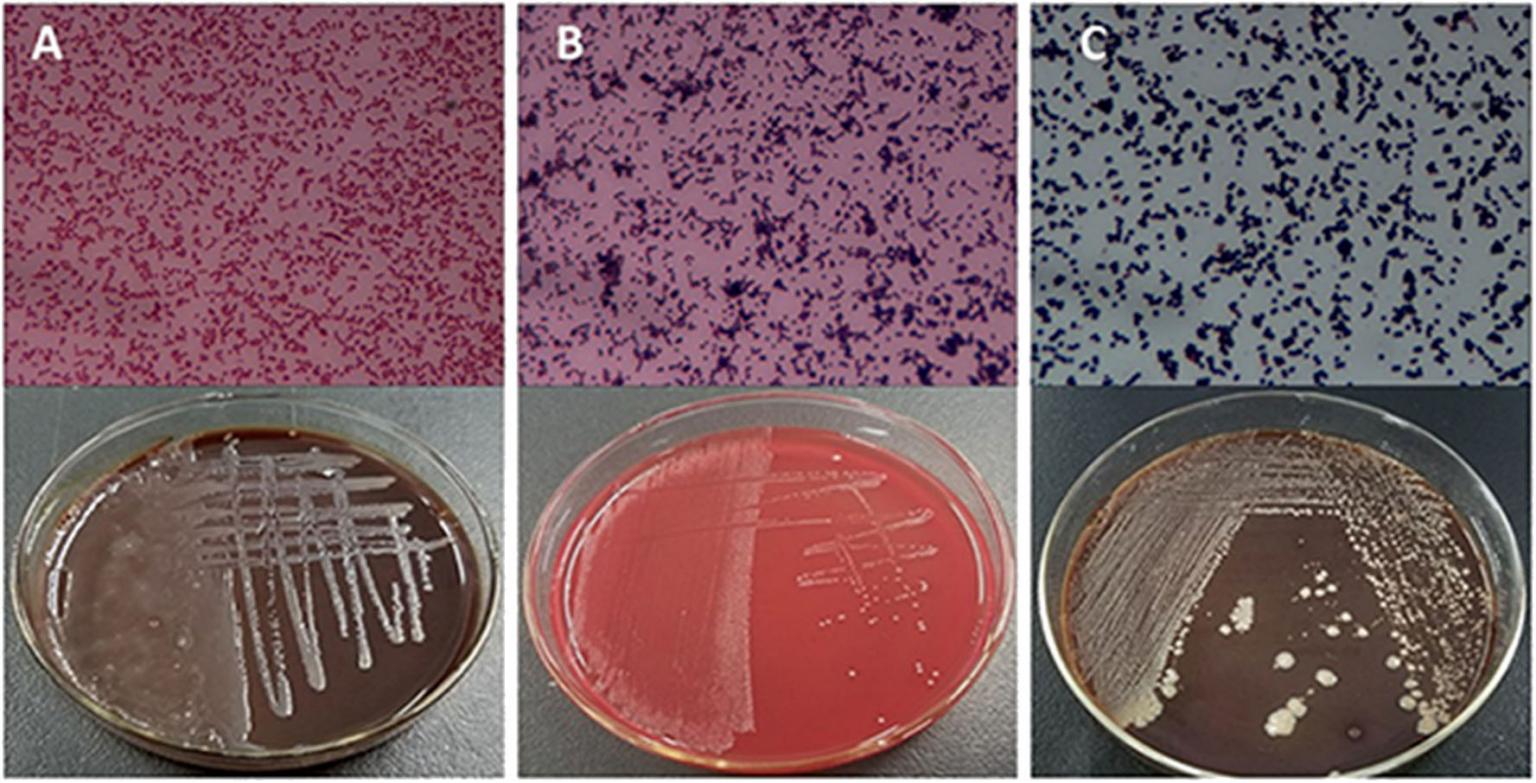
Isolation and culture of pathogenic bacteria of mastitic udders. **(A)** *E. coli*, **(B)** *Streptococcus*, and **(C)** *S. aureus*.

**Figure 2:**
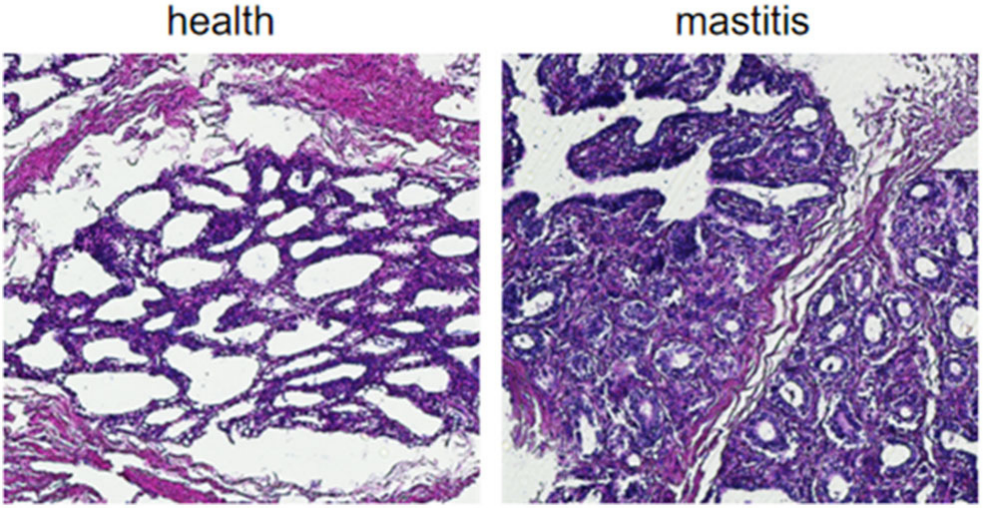
Histological examination of mammary gland tissues of healthy and mastitic cows.

Based on the SCCs, the cows No. 1, No. 4, and No. 8 were initially identified as healthy, and No. 2, No. 6, and No. 7 as mastitic (Table S6). No pathogenic bacteria were isolated from the milk samples of the healthy cows, whereas that from mastitic cows were positive for common mastitis-causing pathogens like *Escherichia coli, Streptococcus*, and *S. aureus* (Figure 1). Furthermore, the mammary glands of healthy cows had closely arranged epithelial cells, and did show any inflammatory cell infiltration in the acinar cavity. However, the mastitic mammary glands showed numerous inflammatory cells and epithelial loss in the acinus cavity (Figure 2). Taken together, No. 1, No. 4, and No. 8 were verified as healthy, and No. 2, No. 6, and No. 7 as mastitic cows.

### Identification of Mastitis-Related miRNAs in Bovine Mammary Glands

**Figure 3:**
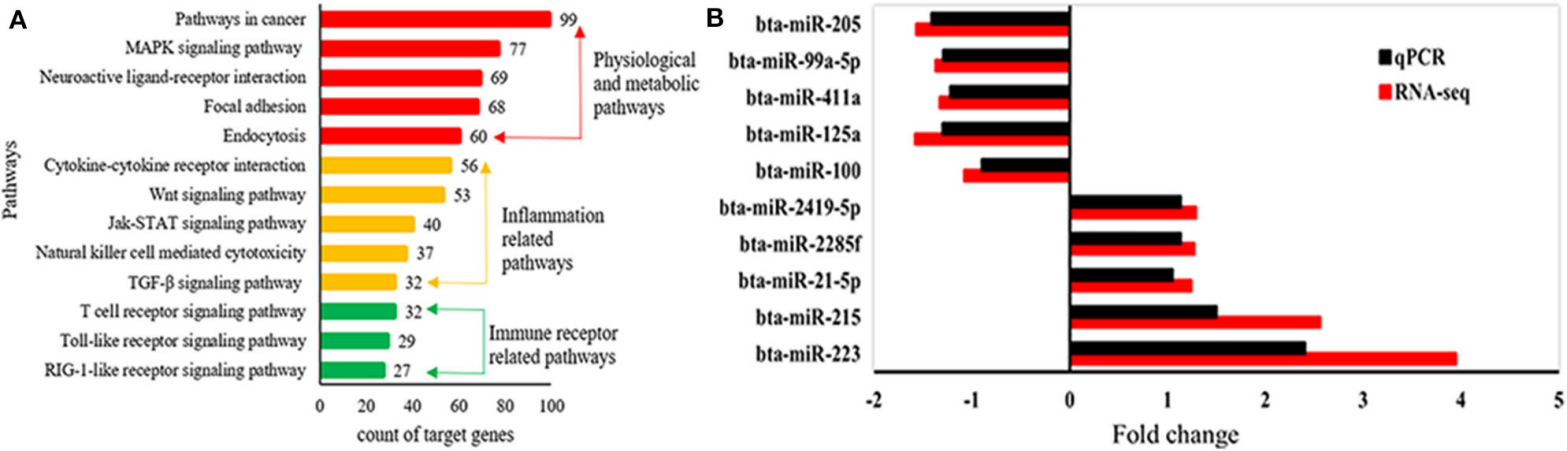
Identification of mastitis-related miRNAs. **(A)** Significantly enriched KEGG pathways of predicted target genes. **(B)** Expression levels of mastitis-related DEMs.

A total of 1,245 miRNAs were identified in the udder tissues of six cows, of which 793 are known (Table S7) and 452 are novel (Table S8). In addition, 465 miRNAs were differentially expressed in the mastitic vs. healthy mammary glands, including 231 up-regulated (143 known and 88 novel) and 234 down-regulated (185 known and 49 novel) miRNAs (Table S9). With |log${}_{2}^{\left(\text{FC}\right)}$| ≥ 1 and FDR ≤ 0.01 as the thresholds, 48 DEMs were statistically significant, including 10 up-regulated and 19 down-regulated known miRNAs (Table 1), and 19 up-regulated novel-miRNAs (Table S10). The DEMs potentially involved in mastitis resistance were screened by functionally annotating their predicted target genes. Inflammation-related pathways were significantly enriched among the target genes (Figure 3A), including pathways in cancer, MAPK signaling pathway, neuroactive ligand-receptor interaction etc. (Table S11). Accordingly, bta-miR-223, bta-miR-205, and bta-miR-21-5p were identified as mastitis resistance-related miRNAs, and also validated by RT-qPCR (Figure 3B).

**Table 1:** Known DEMs in the two libraries.

**miRNA**	**Heath**	**Mastitis**	**FDR**	**${\text{log}}_{2}^{\text{FC}}$**	**Regulated**	**Sig-label**
bta-miR-223	15.51 ± 0.34	170.63 ± 3.46	1.17E-11	3.95	up	^**^
bta-miR-378b	0.22 ± 0.01	1.02 ± 0.02	1.53E-03	2.74	up	^**^
bta-miR-2318	0.76 ± 0.02	2.04 ± 0.34	3.38E-03	2.44	up	^**^
bta-miR-138	1.19 ± 0.03	5.88 ± 0.45	5.14E-03	2.09	up	^**^
bta-miR-21-5p	127451.9 ± 23.41	261332.6 ± 57.32	8.11E-05	1.24	up	^**^
bta-miR-215	43.83 ± 1.32	259.62 ± 6.52	2.02E-03	2.56	up	^**^
bta-miR-2419-5p	14.86 ± 0.69	31.21 ± 1.21	9.97E-03	1.29	up	^**^
bta-miR-2285aa	4.99 ± 0.31	16.74 ± 0.94	2.17E-05	2.25	up	^**^
bta-miR-2285f	87.66 ± 0.18	243.13 ± 2.16	3.91E-03	1.27	up	^**^
bta-miR-2285k	7.92 ± 0.12	21.82 ± 0.36	1.35E-03	1.63	up	^**^
bta-miR-154b	21.16 ± 0.64	13.46 ± 0.34	8.48E-03	−1.38	down	^**^
bta-miR-154c	141.36 ± 5.64	47.49 ± 1.07	1.21E-03	−1.42	down	^**^
bta-miR-199a-3p	9431.34 ± 10.46	4477.21 ± 23.15	1.78E-03	−1.06	down	^**^
bta-miR-204	205.05 ± 2.36	54.16 ± 0.97	3.92E-04	−1.63	down	^**^
bta-miR-205	5529.35 ± 13.45	3086.84 ± 11.69	1.61E-03	−1.57	down	^**^
bta-miR-100	10092.81 ± 84.17	5334.77 ± 57.36	1.30E-03	−1.08	down	^**^
bta-miR-1197	1.3 ± 0.03	0.23 ± 0.01	5.58E-04	−2.61	down	^**^
bta-miR-125a	5829.98 ± 23.22	2063.51 ± 14.57	2.17E-05	−1.58	down	^**^
bta-miR-125b	10753.09 ± 49.51	3792.31 ± 11.53	3.15E-07	−1.68	down	^**^
bta-miR-136	65.42 ± 1.64	34.83 ± 2.09	1.92E-03	−1.45	down	^**^
bta-miR-24	65.64 ± 3.16	20.01 ± 2.17	8.48E-03	−1.26	down	^**^
bta-miR-2299-3p	4.45 ± 0.61	1.24 ± 0.11	2.80E-03	−2.2	down	^**^
bta-miR-380-5p	2.17 ± 0.06	0.23 ± 0.01	1.21E-03	−2.36	down	^**^
bta-miR-380-3p	50.99 ± 3.13	18.54 ± 0.15	3.53E-04	−1.7	down	^**^
bta-miR-411a	2587.08 ± 30.12	1160.6 ± 36.15	2.82E-04	−1.33	down	^**^
bta-miR-411b	25.82 ± 1.08	12.55 ± 1.01	1.91E-03	−1.5	down	^**^
bta-miR-592	6.4 ± 0.11	1.7 ± 0.04	1.45E-03	−2.08	down	^**^
bta-miR-99a-5p	74476.15 ± 63.19	33705.19 ± 79.54	1.49E-05	−1.37	down	^**^
bta-miR-7859	10.42 ± 0.91	2.15 ± 0.03	7.64E-04	−1.94	down	^**^

** *p < 0.01*.

### Bta-miR-223 Mitigates LTA-Stimulated Inflammation in Mac-T Cells by Targeting CBLB and the Downstream Pathway

**Figure 4:**
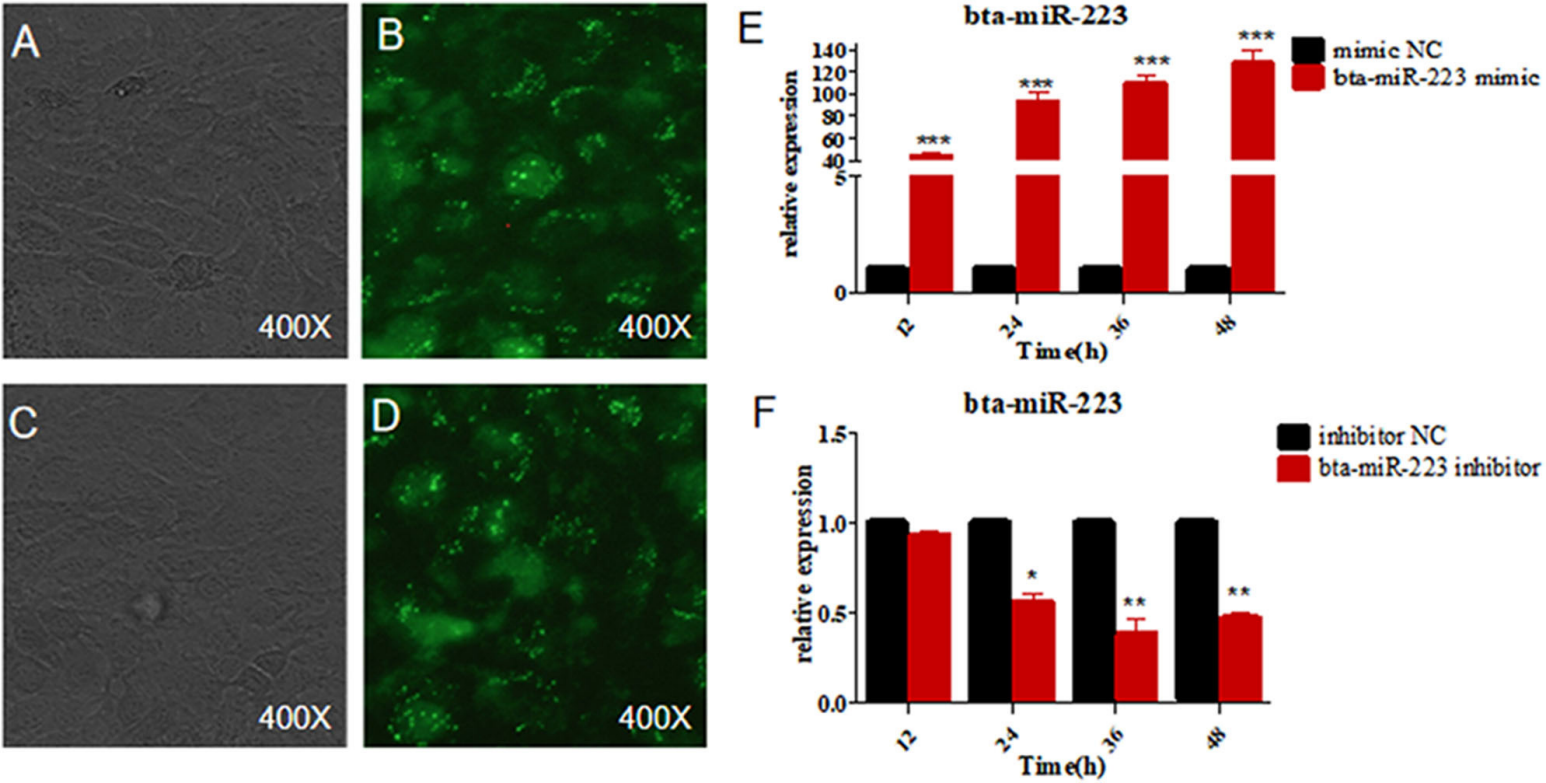
Establishment of bta-miR-223 overexpressing and silenced cell lines. **(A,B)** bta-miR-223 mimic transfected Mac-T cells for 48 h, **(C,D)** bta-miR-223 inhibitor transfected Mac-T cells for 48 h, **(E)** effect of bta-miR-223 mimic on the expression of bta-miR-223, **(F)** effect of bta-miR-223 inhibitor on the expression of bta-miR-223. *p < 0.05, **p < 0.01, ***p < 0.001.

**Figure 5:**
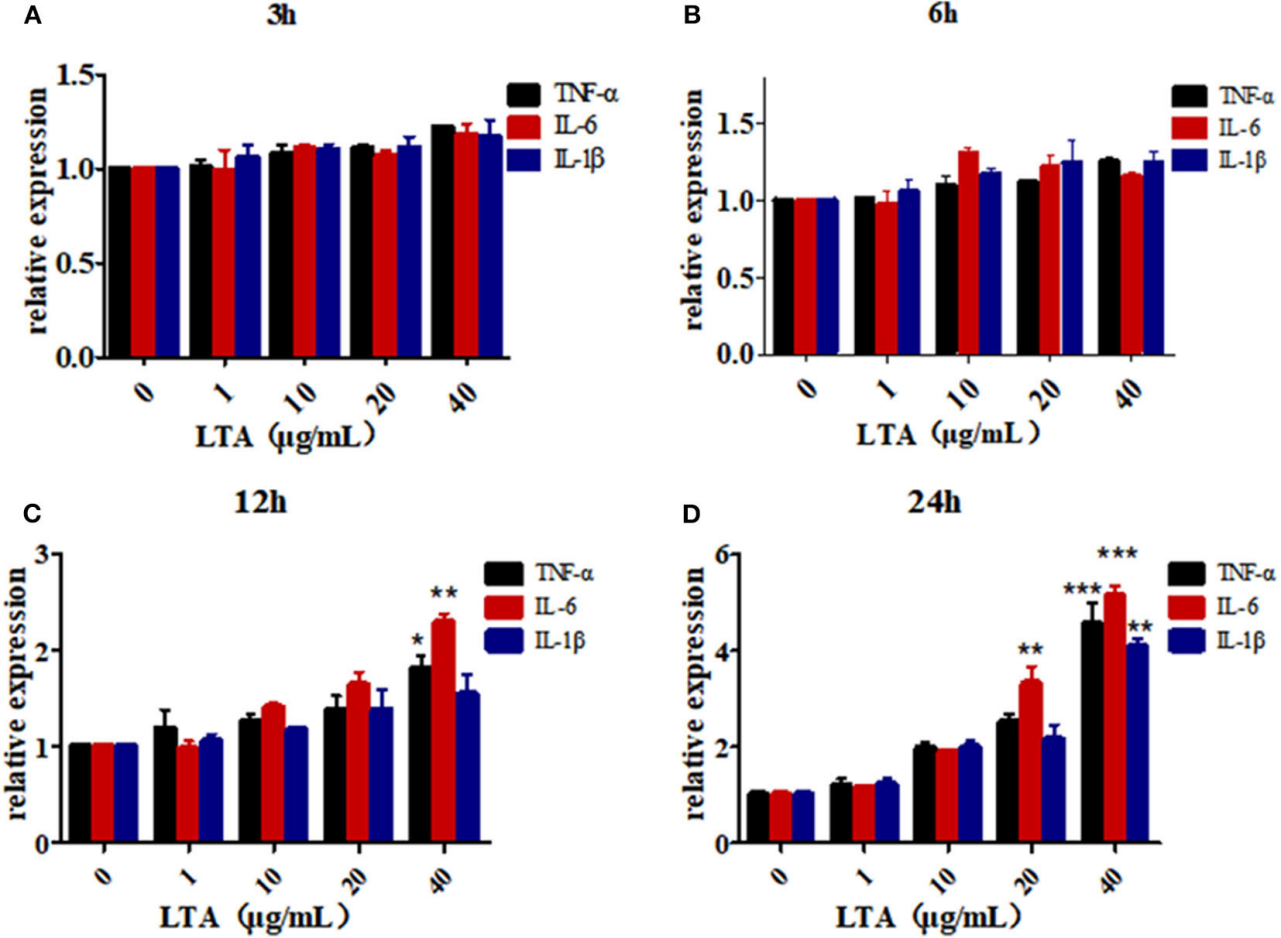
LTA-stimulated inflammation in Mac-T cells. **(A)** LTA stimulates Mac-T cells for 3 h, **(B)** LTA stimulates Mac-T cells for 6 h, **(C)** LTA stimulated Mac-T cells for 12 h, **(D)** LTA stimulated Mac-T cells for 24 h. *p < 0.05, **p < 0.01, ***p < 0.001.

**Figure 6:**
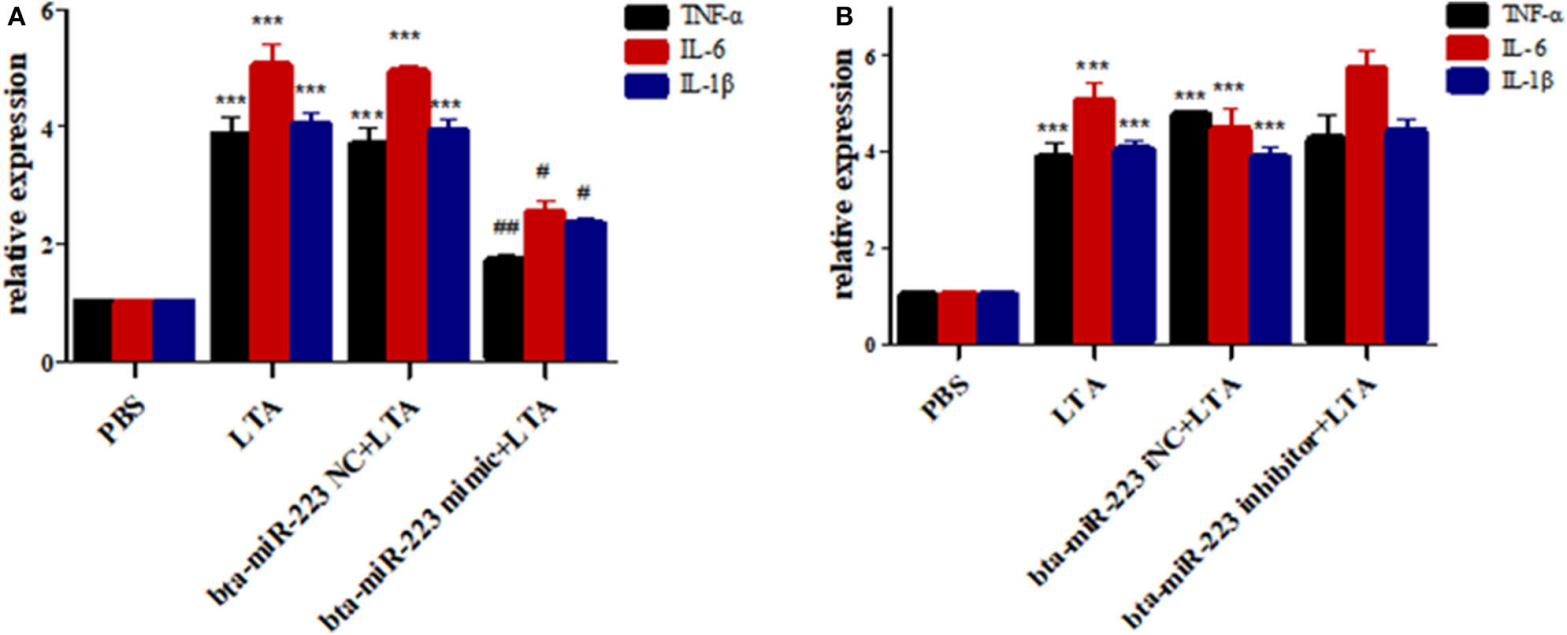
Effect of bta-miR-223 mimic **(A)** and bta-miR-223 inhibitor **(B)** on LTA-stimulated inflammatory factor mRNA expression levels in Mac-T cells. ***p < 0.001 compared to PBS group, ^#^*p* < 0.05 compared to LTA group, ^*##*^*p* < 0.01 compared to LTA groups.

To further determine the role of bta-miR-223 in mastitis, the LTA-stimulated Mac-T cells were transfected with the mimics or inhibitors, and bta-miR-223 expression was successfully enhanced or silenced (Figure 4). The levels of *TNFA, IL6*, and *IL1B* mRNAs in Mac-T cells were unaffected after 3–6 h of LTA exposure, and while *TNFA* and *IL6* were significantly upregulated following 12 h treatment with 40 μg/ml LTA, *IL1B* remained unchanged. However, a 24 h LTA stimulation led to a marked increase in the levels of all three (Figure 5), which was decreased significantly by the bta-miR-223 mimic (Figure 6). Thus, overexpression of bta-miR-223 can mitigate the inflammatory response induced by *S. aureus*-derived LTA *in vitro*, indicating that it likely plays a role in mastitis resistance.

**Figure 7:**
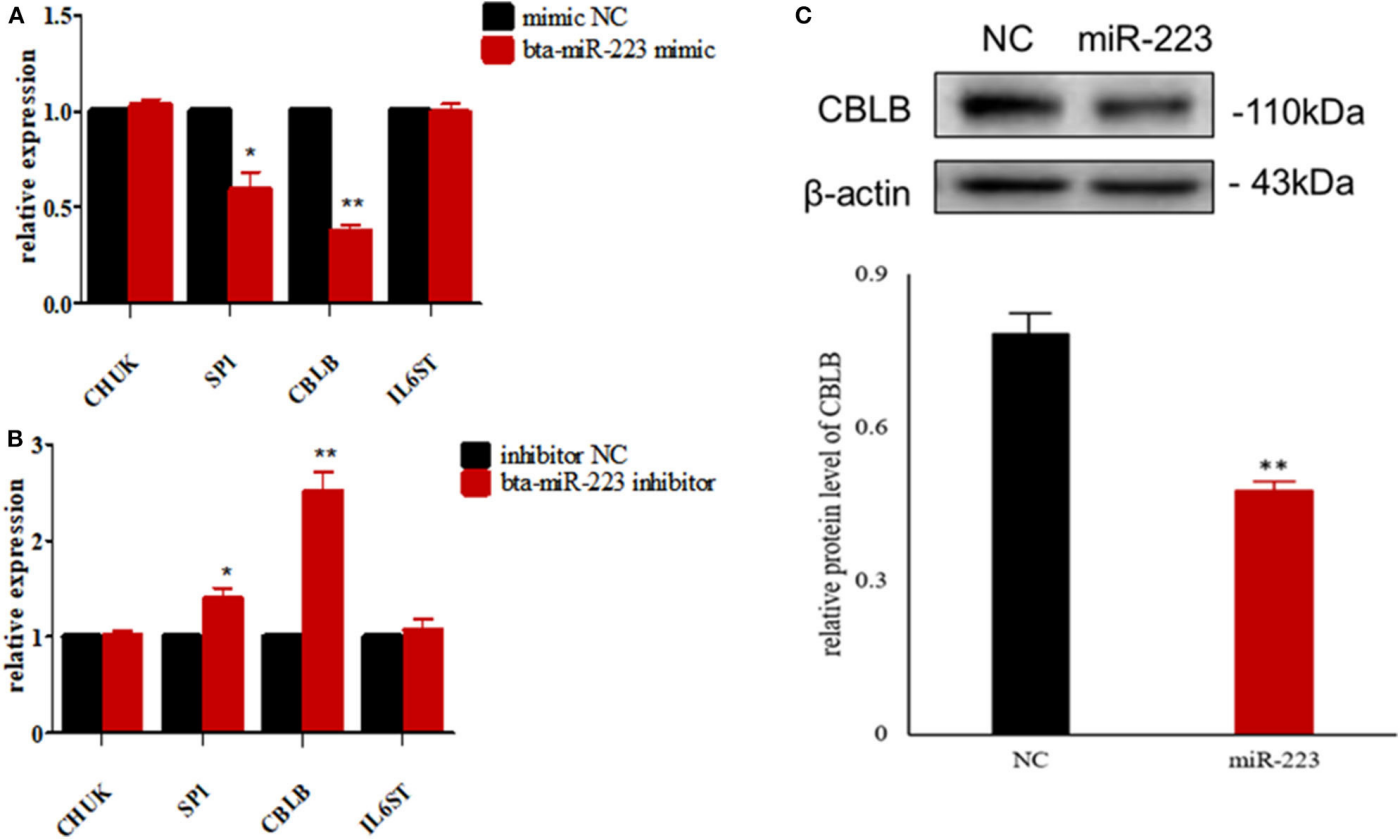
Screening of target genes of bta-miR-223. **(A)** The effect of bta-miR-223 mimic on the mRNA levels of its candidate target genes, **(B)** the effect of bta-miR-223 inhibitor on the mRNA levels of its candidate target genes, **(C)** the effect of bta-miR-223 overexpression on the expression level of CBLB protein. *p < 0.05; **p < 0.01.

**Figure 8:**
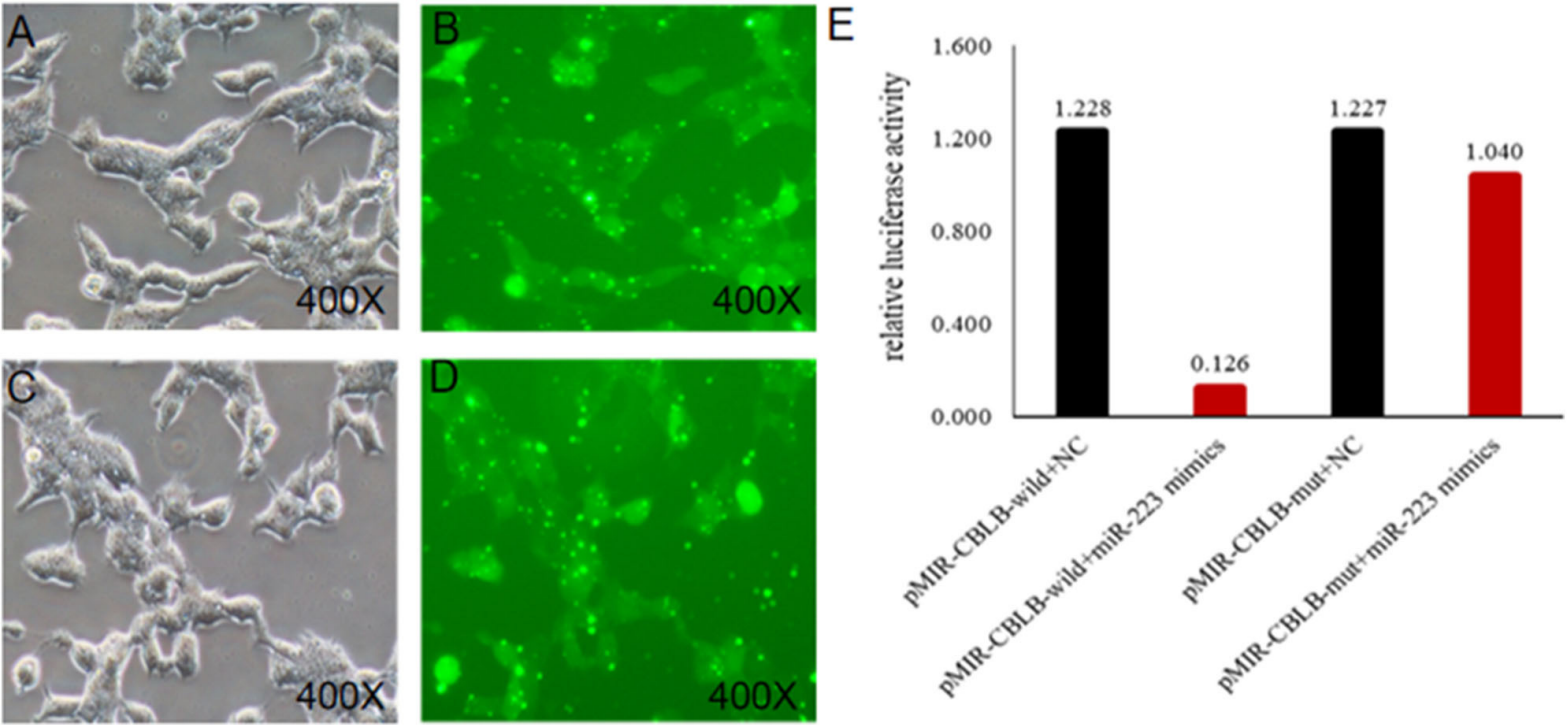
Identification of the targeting relationship between bta-miR-223 and *CBLB*. **(A,B)** bta-miR-223 mimic transfected HEK-293T cells for 48 h, **(C,D)** bta-miR-223 inhibitor transfected HEK-293T cells for 48 h, **(E)** dual luciferase reporter gene test result.

**Figure 9:**
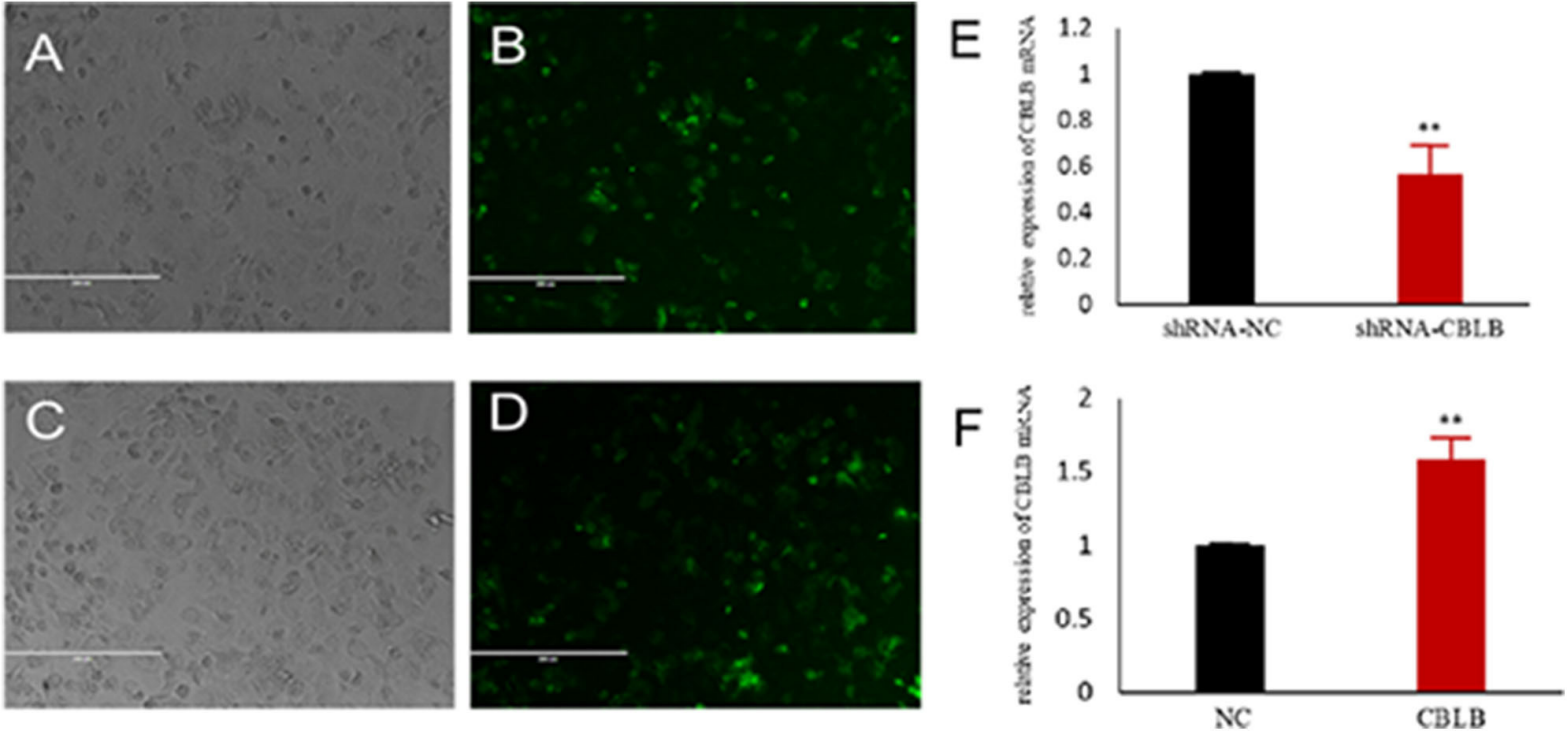
Establishment of CBLB overexpressing and silenced cell lines. **(A,B)** *CBLB* **silenced** vector transfecting into Mac-T cells for 72 h, **(C,D)** *CBLB* **overexpressing** vector transfecting into Mac-T cells for 72 h, **(E)** the mRNA expression of *CBLB* after transfecting *CBLB* **silenced** vector for 72 h, **(F)** the mRNA expression of *CBLB* after transfecting *CBLB* **overexpressing** vector for 72 h. **p < 0.01.

**Figure 10:**
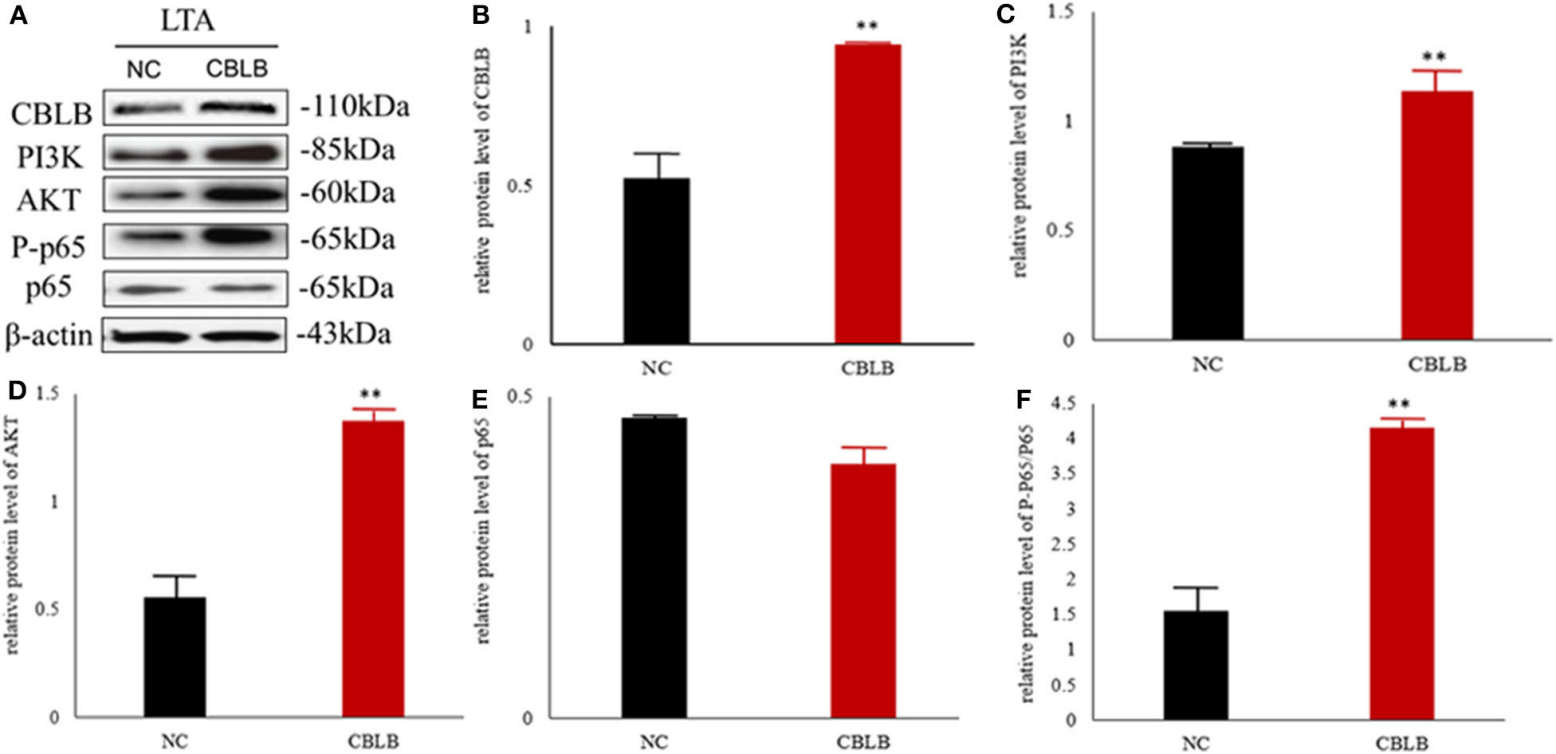
Effect of *CBLB* overexpression on the PI3K/AKT/NF-κB p65 pathway in LTA-stimulated Mac-T cells. **(A)** Immunoblot showing expression levels of CBLB, PI3K, AKT, and NF-κB p65. Quantitative comparison of **(B)** CBLB, **(C)** PI3K, **(D)** AKT, **(E)** NF-κB p65, and **(F)** p-NF-κB p65. **p < 0.01.

**Figure 11:**
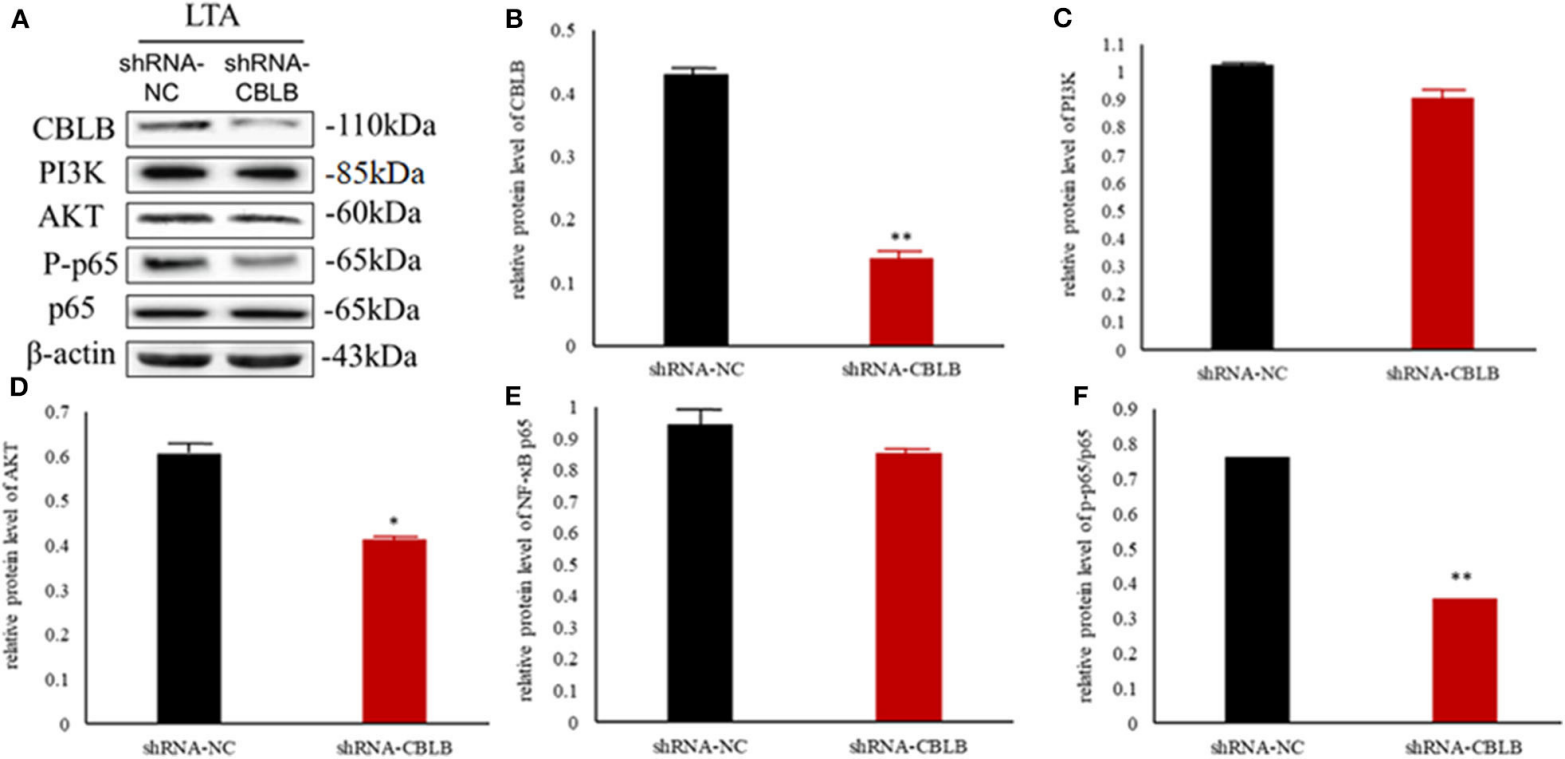
Effect of *CBLB* silence on the PI3K/AKT/NF-κB p65 pathway in LTA-stimulated Mac-T cells. **(A)** Immunoblot showing expression levels of CBLB, PI3K, AKT, and NF-κB p65. Quantitative comparison of **(B)** CBLB, **(C)** PI3K, **(D)** AKT, **(E)** NF-κB p65, and **(F)** p-NF-κB p65. *p < 0.05; **p < 0.01.

According to bioinformatic analyses, *CHUK, SP1, CBLB*, and *IL6ST* were predicted as the target genes of bta-miR-223. Consistent with this, bta-miR-223 overexpression significantly downregulated *SP1* and *CBLB* mRNA in the Mac-T cells whereas bta-miR-223 silencing had the opposite effect. Furthermore, the effect of bta-miR-223 was stronger on *CBLB* compared to *SP1* mRNA. Since *CBLB* protein was also significantly down-regulated by bta-miR-223 mimic (Figure 7), we next determined whether the latter also bound to the *CBLB* promoter through the dual luciferase assay. Briefly, HEK-293T cells were transfected with bta-miR-223 mimic or control with luciferase reporter gene under the wild-type or mutant *CBLB*-3′-UTR, and the relative luciferase activity was measured. As shown in Figure 8, bta-miR-223 mimic sharply decreased the relative fluorescence intensity of wild type but not of mutant *CBLB*-3′-UTR, indicating that bta-miR-223 directly binds to and represses the activity of *CBLB* promoter. The role of *CBLB* in LTA-stimulated inflammation was likewise determined by transfecting the Mac-T cells with the knockdown and overexpression vectors (Figure 9). *CBLB* overexpression in the LTA-stimulated Mac-T cells significantly up-regulated PI3K, AKT, and p-NF-κB p65 (Figure 10), while *CBLB* silencing had an inhibitory effect on their expression levels (Figure 11). Taken together, CBLB drives the *S. aureus* LTA-stimulated inflammatory damage in Mac-T cells via the PI3K/AKT/NF-κB pathway, and is targeted by bta-miR-223.

### *S. aureus*-Induced Mastitis in Mouse Model Is Driven by CBLB

**Figure 12:**
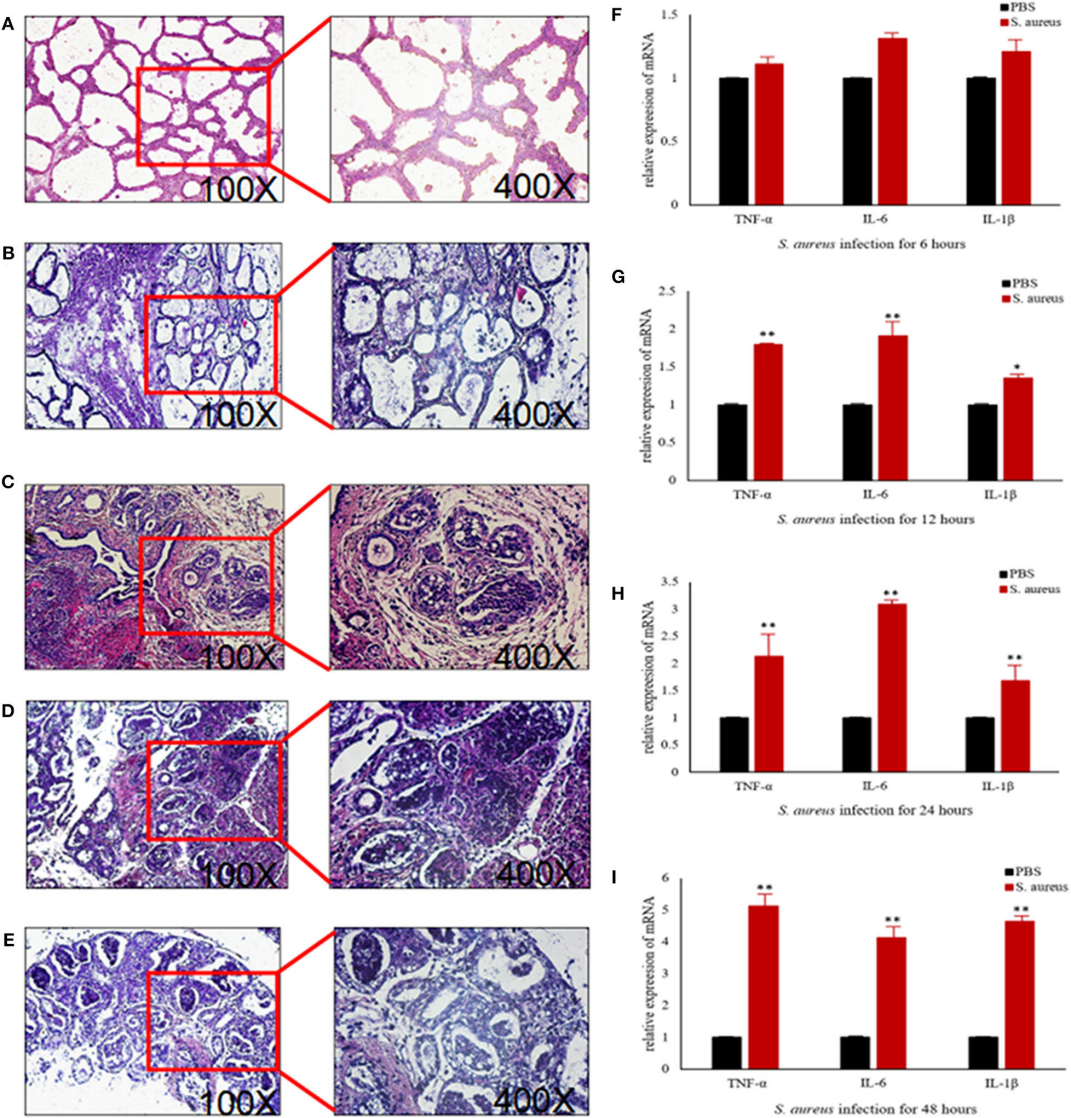
Establishment of *S. aureus*-induced mastitis model in BALB/c mice. Representative images of mammary glands tissue sections of **(A)** PBS control and *S. aureus*-inoculated mice after **(B)** 6 h, **(C)** 12 h, **(D)** 24 h, and **(E)** 48 h after infection. Expression levels of pro-inflammatory cytokine mRNAs in the mammary glands after **(F)** 6 h, **(G)** 12 h, **(H)** 24 h, and **(I)** 48 h after infection. *p < 0.05; **p < 0.01.

**Figure 13:**
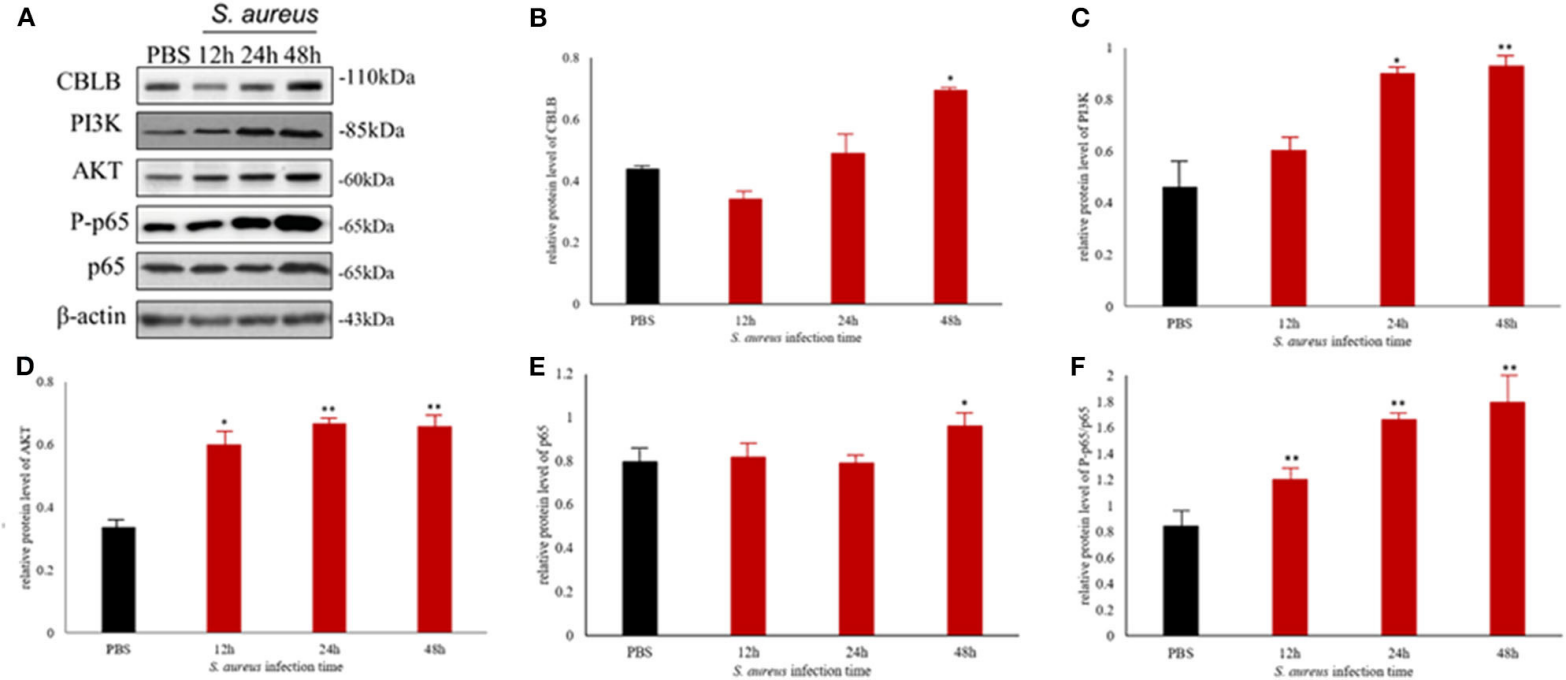
The CBLB/PI3K/AKT/NF-κB p65 pathway is activated in the *S. aureus*-infected mammary gland tissues of BALB/c mice. **(A)** Immunoblot showing expression levels of CBLB, PI3K, AKT, and NF-κB p65. Quantitative comparison of **(B)** CBLB, **(C)** PI3K, **(D)** AKT, **(E)** NF-κB p65, and **(F)** p-NF-κB p65. *p < 0.05; **p < 0.01.

To further explore the above hypothesis, we established a mouse model of *S. aureus*-induced mastitis by injecting a high bacterial titer directly into the mammary glands of postpartum mice. Compared to intact tissue structure, closely arranged epithelial cells, and lack of inflammatory cells in the acinar cavity of the mammary glands of PBS-injected controls, the *S. aureus*-infected glands showed a time-dependent increase in inflammation and tissue damage. The inflammatory cells started to infiltrate into the acinar cavity within 6 h of *S. aureus* inoculation, and increased over the course of infection. The mammary tissue structure was largely intact at the 6 h time point and started to collapse by 12 h with epithelial cell depletion and massive infiltration of the inflammatory cells. These pathological changes steadily worsened till 48 after the infection. Consistent with the inflammatory damage, *TNFA, IL6*, and *IL1B* mRNA levels started to spike 12 h after *S. aureus* infection, and increased steadily till 48 h (Figure 12). Furthermore, the infected glands showed significantly higher levels of p-NF-κB p65 after 12 h compared to the uninfected control, while PI3K, AKT, and p-NF-κB p65 were upregulated after 24 h. Interestingly, CBLB protein levels increased significantly only after 48 h of infection, wherein all the aforementioned proteins were also upregulated (Figure 13). These results clearly indicate that CBLB plays a key role in driving *S. aureus*-induced inflammatory damage in mammary glands by activating the PI3K/AKT/NF-κB pathway.

## Discussion

Bovine bacterial mastitis-related miRNAs were first identified by Jin et al. in Mac-T cells infected with heat-inactivated *S. aureus* and *E. coli*. A total of 231 known and 113 novel-miRNAs were identified in that study, including miR-21-5p, miR-27b, miR-22-3p, miR-184, let-7f, miR-2339, miR-499, miR-23a, and miR-99b that were specific to the *S. aureus*-infected cells (8). More recently, Luoreng et al. identified 279 and 305 DEMs in the mammary tissues of cows infected with *S. aureus* and *E. coli*, respectively (13). Furthermore, 221 known and 69 novel miRNAs have been identified in the milk exosomes of *S. aureus*-infected cows, of which 22 and 15, respectively showed differential expression compared to that in healthy cows (22). In this study, we identified 48 DEMs in the udders of mastitic cows, including 10 up-regulated and 19 down-regulated known miRNAs and 19 up-regulated novel-miRNAs. Bta-miR-223 and miR-21-5p were in particular significantly up-regulated, while bta-miR-205 showed a marked reduction in the mastitic vs. the healthy mammary gland tissues. Consistent with this, Fang et al. also detected a significant increase in the expression levels of bta-miR-223 and bta-miR-21-3p following infection with a high titer of *S. aureus* (10). Another study reported that miR-223 was up-regulated and miR-205 was down-regulated in the mammary glands of *S. aureus*-infected cows (9). In addition, miR-223, miR-9, miR-125b, miR-155, and miR-146a were highly expressed in bovine CD14^+^ monocytes stimulated with lipopolysaccharide or *S. aureus*-derived enterotoxin B (23).

Since the putative target genes of bta-miR-223 were functionally annotated to pathways in cancer, MAPK signaling, neuroactive ligand-receptor interactions, endocytosis, cytokine-cytokine receptor interactions signaling, we hypothesized that this miRNA likely controls the inflammatory responses involved in mastitis. Indeed, miR-223 is significantly up-regulated in the colonic mucosa of patients with ulcerative colitis (24) and active inflammatory bowel disease (25), the thymus, lung and liver of fetuses during acute chorioamnionitis (26), T cells and lymphoid tissues of arthritic patients (27), *S. aureus*-infected wounds (28), and bovine endometriotic tissues (29). Furthermore, overexpression of miR-223 in various *in vitro* and *in vivo* systems increased resistance to *S. aureus* infection (25), blocked NLRP3 inflammasome activation and IL1B production (29), inhibited the TLR4/NF-κB signaling pathway (30), reduced macrophage foam cell formation, lipid accumulation, and pro-inflammatory cytokine production in atherosclerotic lesions (31), down-regulated IL6 secretion from monocytes (32), and repressed IL6 and TNFA expression (33). In agreement with these studies, we found that overexpression of bta-miR-223 in Mac-T cells decreased the expression levels of pro-inflammatory cytokines, indicating that it can mitigate the LTA-stimulated inflammatory response *in vitro*.

To further explore the mechanistic basis of the anti-inflammatory effects of bta-miR-223, we predicted several target genes *in silico*, and subsequently identified *CBLB* as the direct target through functional validation. CBLB is an E3 ubiquitin-protein ligase that tags proteins for proteosome-mediated degradation (34). The deletion or inactivation of CBLB could make natural killer cells spontaneously resist metastatic tumors (35). CBLB-deficient mutations mice could develop a certain degree of spontaneous malignant tumor resistance (36, 37), could spontaneously reject tumor cells which expressing human papilloma virus Ags (38), showed a high survival rate during fatal systemic infection (39). However, little is known regarding the role of *CBLB* in anti-bacterial immunity. We found that *CBLB* overexpression augmented the levels of PI3K, AKT, and p-NF-κB p65 following LTA stimulation, while its knockdown had the opposite effects. The NF-κB pathway plays a key role in mediating the inflammatory and innate immune responses, and its constitutive activation in various autoimmune diseases triggers cell death and extensive tissue damage (40). Therefore, our findings strongly suggest that *CBLB* promotes the inflammatory damage in mastitis via the PI3K/AKT/NF-κB pathway.

To validate the above hypothesis, we established an *in vivo* model of mastitis by injecting *S. aureus* into the mammary glands of BALB/c mice. After 48 h of infection, the levels of inflammatory cytokines in the acinar cavity increased significantly and was accompanied by extensive tissue damage, along with upregulation of CBLB, PI3K, AKT, and p-NF-κB p65 in the mammary glands. Since bta-miR-223 was up-regulated in the mastitic bovine mammary glands, we expected a downregulation in CBLB in the *S. aureus*-infected glands of mice. However, CBLB protein levels did not change significantly during the 48 h infection period and only increased thereafter, whereas PI3K, AKT, and p-NF-κB p65 were upregulated at earlier time points. This apparent discrepancy can be attributed to the temporal changes in the host immune response to bacterial infection. In the first 6 h of infection, *S. aureus* expressed multiple genes related to adhesion, invasion and host defense to escape the immune system (41), which corresponded to the absence of an inflammatory response in the mammary gland. The subsequent increase in the production of TNFA and other pro-inflammatory cytokines between 12 and 24 h of infection increased the expression levels of miR-223 (42), which in turn repressed *CBLB* expression. However, sustained *S. aureus* proliferation over the 48 h following infection likely led to a severe inflammatory response that destroyed the mammary gland tissues, and released degrading enzymes or oligonucleotides that degraded bta-miR-223 and reversed the negative regulation of *CBLB*, eventually increasing its protein levels. To summarize, *S. aureus* initiates an immune escape mechanism in the early stages of infection to colonize the udder, and triggers an inflammatory response that upregulates bta-miR-223 and prevents CBLB expression. However, protracted inflammation eventually overcomes the repressive effect of bta-miR-233 on CBLB. The latter augments the levels of PI3K, AKT, and p-NF-κB p65 by ubiquitinating specific proteins and further promotes the inflammatory pathway.

In summary, we identified 29 up-regulated and 19 downregulated miRNAs in the mastitic udders, of which bta-miR-223, bta-miR-205, and bta-miR-21-5p are involved in multiple inflammatory response-related signaling pathways. In addition, bta-miR-223 likely down-regulated the inflammatory response in the LTA-stimulated Mac-T cells by targeting CBLB, indicating that it is involved in the host immune response against *S. aureus*-induced mastitis.

## Conclusion

Bta-miR-223 inhibits the PI3K/AKT/NF-κB inflammatory pathway in LTA-stimulated Mac-T cells by directly targeting *CBLB*.

## Data Availability Statement

The datasets presented in this study can be found in online repositories. The names of the repository/repositories and accession number(s) can be found below: https://www.ncbi.nlm.nih.gov/bioproject/PRJNA504808/.

## Ethics Statement

The animal study was reviewed and approved by the Animal Protection Committee of Heilongjiang Bayi Agricultural University. Written informed consent was obtained from the owners for the participation of their animals in this study.

## Author Contributions

SH and BS contributed to conception and design of the study. SH, XL, JL, ZZo, and LL performed the statistical analysis. SH wrote the first draft of the manuscript. All authors contributed to manuscript revision, read, and approved the submitted version.

## Conflict of Interest

The authors declare that the research was conducted in the absence of any commercial or financial relationships that could be construed as a potential conflict of interest.

## Supplementary Material

Table S1 Reverse transcription primer sequences for bovine miRNAs.

Table S2 RT-qPCR primer sequences for bovine miRNAs.

Table S3 RT-qPCR primer sequences for bovine inflammatory cytokines.

Table S4 RT-qPCR primer sequences for miR-223 target genes.

Table S5 RT-qPCR primer sequences for murine inflammatory cytokines.

Table S6 Somatic cell counts in milk from healthy and mastitic cows.

Table S7 Sequences of known miRNAs.

Table S8 Sequences of novel miRNAs.

Table S9 Expression levels of miRNAs in the mammary gland tissues.

Table S10 Novel differentially expressed miRNAs in the two libraries.

Table S11 KEGG annotation of the target genes of differentially expressed miRNAs.
